# Physical Activity as a Lifestyle Intervention for Type 1 Diabetics: A Comprehensive Review

**DOI:** 10.7759/cureus.87351

**Published:** 2025-07-05

**Authors:** Noha A Alzahrani, Ihdaa J Abdulwahab, Moteab S Alotaybi, Abdulaziz Albogami

**Affiliations:** 1 Preventive Medicine, Ministry of National Guard-Health Affairs, Jeddah, SAU; 2 Preventive Medicine, Ministry of National Guard, Jeddah, SAU; 3 Faculty of Dentistry, King Abdulaziz University, Jeddah, SAU

**Keywords:** body weight, exercise, lifestyle medicine, thinness, type 1 diabetes mellitus

## Abstract

The aim of this study was to assess the influence of aerobic and resistance training on anthropometric measures such as body mass index, body weight, fat mass, and fat-free mass among type 1 diabetics. A thorough literature review was performed on PubMed. Our search yielded 196 articles. We included eight studies that dealt with type 1 diabetes mellitus (T1DM) patients who met our inclusion criteria and consisted of randomized controlled trials, systematic reviews, and meta-analyses.

This review article found that resistance exercise increased muscle mass and reduced fat mass in T1DM patients. Increasing the duration and intensity of physical activity led to an increase in fat-free mass. Parental support among type 1 diabetic children showed positive attitudes towards a healthy lifestyle. Addressing stigma among adolescents with T1DM is important, and personal and community knowledge about the condition can help facilitate physical activity participation. Collaboration between healthcare providers, families, and peers is essential in managing physical activity in T1DM patients. Having a staff member with specific training to provide physical activity advice can benefit diabetes teams. Further research over a longer duration is necessary to confirm these outcomes.

## Introduction and background

The prevalence of type 1 diabetes mellitus (T1DM) was estimated in 2021 to be around 8.4 million individuals worldwide [1]. Myopathy is one of the common clinical in T1DM patients, characterized by a decrease in muscle mass and function [2]. The benefits of physical activity (PA) are exclusive not only to healthy people but also to T1DM patients. Hence, if PA was combined with proper nutrition, it would form the basic treatment for diabetes [3]. Any movement that increases energy expenditure is considered PA, and if it is planned and structured, it is exercise [4,5]. Moreover, T1DM patients can benefit from regular exercise, which improves cardiovascular fitness, muscle strength, and insulin sensitivity [6].

Training depends primarily on intensity and duration and whether it is aerobic or anaerobic. Aerobic exercise consists of engaging large groups of muscles for a long period of time with moderate intensity to maintain a high heart rate while using oxygen to produce ATP, for example, walking, jogging, cycling, or swimming. On the other hand, anaerobic exercise goal is to increase muscle strength, power, and speed through resistance training or any activity performed as sprint form such as running or cycling [7,8].

Aerobic exercise has been shown to positively affect body weight, cardiorespiratory fitness, and cardiac output in diabetics [9,10], while resistance exercises have a beneficial effect on body composition, muscle mass, and bone density [11,12].

The aim of the review was to assess the influence of aerobic and resistance training on anthropometric measures such as body mass index (BMI), body weight, fat mass, and fat-free mass among type 1 diabetics. This review also concluded with a recommendation for behavior change in a lifestyle medicine clinic.

## Review

Methodology

Search Strategy

A PubMed literature search was conducted using the following terms:("type 1 diabetes" OR "T1DM") AND ("physical activity" OR "exercise") AND ("thinness" OR "low body mass index" OR "low BMI") AND ("resistance training" OR "sport") AND ("physical endurance"). Our search was filtered by title, abstract, full-text availability, English studies only, and the last 10 years of publication between 2014 and March 2024. Our search yielded 196 articles. We included eight studies that dealt with T1DM patients who matched our inclusion criteria and consisted of randomized controlled trials (RCTs) and systematic reviews and meta-analyses.

Inclusion and Exclusion Criteria

All possible studies were considered eligible if they matched the following criteria: 1) a patient with T1DM, 2) any age group, 3) covered at least one component of PA intervention, and 4) included at least one anthropometric measurement of body weight, BMI, fat-free mass, or fat mass as a primary or secondary outcome. The studies were excluded if they did not involve T1DM patients, were not intervention trials, or did not use anthropometric measures as an endpoint.

Review

This is the first review article to examine anthropometric outcomes after PA intervention among T1DM patients. Research on underweight patients with T1DM is limited in terms of determining the most effective method for healthy weight gain.

Table 1 shows study characteristics such as design, duration, and participation characteristics, including age, gender, sample size, and PA type. Table 1 illustrates two studies in Poland [3,13] and one in each of the following countries: Belgium [14], Brazil [15], Australia [16], New Zealand [17], Canada [6], and the UK [12]. Out of the eight reviewed studies, three were RCTs [3,13,17], one was a non-RCT [15], one was a systematic review of an RCT [14], and three were systematic reviews and meta-analyses of RCTs [6,12,16]. The study duration ranged from 6 weeks to 20 weeks. For all the included studies, the participants were children, adolescents, and adults of both genders. The sample size varied from 16 to 756. The interventions were either aerobic, resistance exercise, or both combined in one arm.

**Table 1:** Characteristics and participants of the included studies (n = 8) EG, experimental group; CG, control group; NR, not reported; PA, physical activity; RCT, randomized controlled trial

Author, year, country	Study characteristics and participants
Wróbel et al. (2018) [3], Poland	Design and duration: RCT, 3 months
	Age and gender: adult: 30-45 years, male
	Sample size: n = 21, EG1 = 11, EG2 = 11
	Type of PA: aerobic and resistance training
Yardley et al. (2014) [6], Canada	Design and duration: systematic review and meta-analysis of RCT (4)
	Age and gender: 15-50 years, male and female
	Sample size: n = 323, EG = 200, CG = 95
	Type of PA: aerobic and resistance training
Quirk et al. (2014) [12], the UK	Design and duration: systematic review and meta-analysis of RCT and non-RCT (23)
	Age and gender: ≤18 years, boys and girls
	Sample size: n = 756
	Type of PA: aerobic and resistance training
Wróbel et al. (2021) [13] Poland	Design and duration: RCT, 6 weeks
	Age and gender: 30–45 years, men
	Sample size: n = 16, EG = 8, CG = 8
	Type of PA: aerobic and resistance training
Absil et al. (2019) [14], Belgium	Design and duration: systematic review of RCT studies, n = 7, 12-24 weeks
	Age and gender: 13-16 years, boys and girls
	Sample size: n = 361
	Type of PA: aerobic and anaerobic training
Farinha et al. (2018) [15], Brazil	Design and duration: non-RCT, parallel design, open-label trial, 10 weeks
	Age and gender: adult :18-40 years, male and female
	Sample size: n = 28, EG1 = 9, EG2 = 9, EG3 = 10
	Type of PA: HIIT and/or ST
Ostman et al. (2017) [16], Australia	Design and duration: systematic review and meta-analysis of RCT (15), average time for intervention 18.7 weeks
	Age and gender: adult and children, male and female
	Sample size: n = 596, EG = 360, CG = 236
	Type of PA: all types of PA
Gusso et al. (2016) [17], New Zealand	Design and duration: RCT, 20 weeks
	Age and gender: adolescents: 14-18 years, boys and girls
	Sample size: n = 75, EG1 = 38, CG1 = 15, CG2 = 22
	Type of PA: aerobic and resistance training

Intervention Aspects

Table 2 presents the interventions and primary findings of the research. All studies provided participants with instructions regarding the intervention plan, which may be either aerobic, strength-based, or a combination of both. Several of the trials incorporated supervised intervention. Various instruments were employed to monitor the heart rate. Most of the participants participated in organized sessions that consisted of a warm-up, either an aerobic or resistance workout, and a cool-down, which concluded with stretching. One study involved conducting the intervention under both hypoxic and normoxic conditions [13].

**Table 2:** Interventions and main findings of the studies 1RM, maximum weight that can be lifted for one repetition; 10RM, maximum weight that can be lifted for 10 repetitions; AG, aerobic group; BFM, body fat mass; ES, effect size; FiO_2_, fractional concentration of inspired oxygen; HC, healthy controls who trained; HIIT, high-intensity interval training; HRmax, cardiopulmonary exercise test's maximum heart rate; HRR, heart rate reserve; MD, mean difference; NS, not significant; RG, resistance group; SMD, standardized mean difference; SMM, skeletal muscle mass; ST, strength training; T1DM, type 1 diabetes mellitus; T1DM+C, T1DM patients who did not train; T1DM+I, T1DM patients who trained; WHR, waist-to-hip ratio

Author, year country	Intervention components	Main findings
Wróbel et al. (2018) [3], Poland	The electrical impedance method (Inbody 720, Biospace Co., Tokyo, Japan) was used to measure body mass and composition at baseline and after a 3-month intervention in the morning (7:00–8:00 am) after an overnight fast. Food and drink were observed the night before these assessments.	Body weight (kg): RG (MD=0.2, P>0.05), AG (MD=0.1, P>0.05)
	Aerobic group: each training session had a 5-minute warm-up, 50-minute main part, and a 5-minute finish. The main workout intensity was 75% of the lactate threshold workload from the endurance test.	BMI (kg/m²): RG (MD=0, P>0.05), AG (MD=0.2, P>0.05)
	Resistance training group: participants in this group chose their own intensity levels. The experiment had two sessions.	Muscle mass (kg): RG (MD=0.7, P>0.05), AG (MD=0, P>0.05)
	In session 1, the goal was to determine the 1RM for leg extension, lying leg curl, barbell upright row, wide-grip pulldown, and chest press.	Fat mass (kg): RG (MD=-0.9, P>0.05), AG (MD=0.7, P>0.05)
	Session 2 had two weekly training sessions. The load increased with each training session. The load increased by 2.5% per workout. Every set was performed until muscular failure.	–
Yardley et al. (2014) [6], Canada	In the studies, interventions included either aerobics, strength, or both types of exercise. Intensity ranges from 65% to 90% HRR	Body weight (kg): MD=1.1; 95% CI: 0.11, 2.1; P=0.03 (43 participants)
	There was a total of 295 participants, with 200 in the exercise groups and 95 in the control groups.	BMI (kg/m²): MD=-0.02; 95% CI: -0.04, 0.37; P=0.93 (265 participants)
	The studies varied in length, ranging from 3 to 9 months. The number of exercise sessions per week ranged from 2 to 5 per week. The duration of each session ranged from 30 to 120 minutes per session	–
Quirk et al. (2014) [12], UK	The duration of interventions ranged from 2 to 39 weeks.	BMI (kg/m²): SMD=-0.41; 95% CI: -0.7, -0.12; P=0.006 (195 participants)
	The interventions were aerobic, a mix of aerobic and strengthening activities, or balance and flexibility.	The meta-analysis was unable to pool the results of five research.
	Activity sessions lasted between 30 and 120 minutes. The frequency of sessions varies from 1-5 days per week.	Salem et al. [18] saw a notable decrease in BMI and waist circumference in children who participated in physical activity sessions once and three times a week, with a more pronounced impact shown in those who engaged in thrice-weekly sessions.
	Aerobic activities intensity was carried out at light (55-64% HRmax), moderate-to-vigorous (65-74% HRmax), vigorous (75-90% HRmax), or a combination of moderate and vigorous intensity.	According to Sideraviciuta et al. [19], teenagers diagnosed with T1DM exhibited an elevated body mass index (BMI) both before and after participating in a swimming program. There was a decrease in adipose tissue mass following the intervention.
	The activity intensity: strengthening activities were done based on 1RM, 10RM, and 85-95% HRmax.	The group engaged in physical activity [20] showed an increase in fat-free mass, which was also positively associated with the duration of moderate, vigorous, and moderate-to-vigorous physical activity [21].
	–	Following a 12-week three-times-weekly circuit exercise program, adolescent males diagnosed with T1DM showed an increase in lean body mass and a reduction in fat mass.
Wróbel et al. (2021) [13], Poland	Two groups have been randomly assigned to train	Anthropometric measurements
	Normoxic conditions: standard indoor air with a FiO_2_=20.9%	There were no differences among groups between baseline and after 6 weeks of combined training in terms of body weight, BMI, WHR, fat mass, and muscle mass.
	Hypoxia conditions: in a hypoxia chamber, equivalent to an altitude of 2,500 m above sea level with FiO_2_ = 15.4%.	Weight (kg): hypoxia: at baseline (89.7 ± 16.5), in the 6th week (89.6 ± 16.7). Normoxia: at baseline (94.1 ± 6.4), in the 6th week (93.6 ± 6.3) (P>0.05).
	Both groups: normoxic conditions group and altitude hypoxia group (normobaric) follow same protocol of warm-up, aerobic, and resistant exercise.	BFM (kg): hypoxia: at baseline (22 ± 4.6), in the 6th week (21.8 ± 5.12). Normoxia: at baseline (22.8 ± 6.6), in the 6th week (22.6 ± 7.6) (P>0.05).
	Training sessions: 2 sessions per week, each 60 minutes long, for 6 weeks.	SMM (kg): hypoxia: at baseline (38 ± 7.5), in the 6th week (38 + 7.6). Normoxia: at baseline (40.4 ± 2.4), in the 6th week (40.3 ± 2.6) (P>0.05).
	Warm-up: each training session started by 5-minute general warm up, followed by 15-minute warm up of the upper body, shoulders, and push-ups. During warm- up, they performed 15 repetitions at 20% of their estimated 1RM followed by 10 repetitions at 40% 1RM.	BMI (kg/m²): Hypoxia: at baseline (27.1 ± 2.7), in the 6th week (27.0 ± 2.8). Normoxia: at baseline (28.2 ± 1.5), in the 6th week (28.1 ± 1.5) (P>0.05).
	Aerobic: each session includes treadmill aerobics for 10 sets of 1 minute in combination with resistance exercise for 10 repetitions at 50% 1RM load. Gradual increase from 50% to 75% HRmax, about 5% weekly.	–
	Resistance: two exercises include barbell bench press and barbell front raise. Gradual load increases by 2.5 kg weekly.	–
Absil et al. (2019) [14], Belgium	Regarding the type of exercise, four studies combined aerobic and anaerobic physical activities, and three studies evaluated only aerobic exercise.	Six studies out of seven mentioned anthropometrics measurement in outcome. Of those, 3 only had significant results after the intervention was applied on T1DM patients.
	The aerobic activities corresponded to walking and running outside or on a treadmill, stepping, and cycling, while the anaerobic ones consisted in strengthening exercises, circuit and interval training, workloads and balance exercises.	Heyman et al. [20] and Roberts et al. [22] observed a substantial rise in the average height of the patients after 24 weeks, which may be attributed only to their growth progression.
	Workload varied between low-to-moderate intensity to moderate-to vigorous intensity physical activity.	The studies conducted by Heyman et al. [20] and Salem et al, [18] both reported a substantial rise in overall weight. This could be attributed to either the substantial rise in FFM and FM associated with growth, or to the development of muscle mass.
	In four studies, the training sessions were supervised by educators or physiotherapists. In another study, sessions were at first supervised by professionals for 12 weeks, and then the patients were invited to maintain their physical activity unsupervised for 12 additional weeks,	The study conducted by Salem et al. [18] reported a decrease in waist circumference among the groups receiving the intervention. This finding implies that the notable rise in BMI they found was not associated with an increase in flat fat mass.
	On average, patients practiced 144.3 ± 43.0 minutes of physical activity per week during the programs, in a range from 60 to 180 minutes. The frequency of exercise sessions fluctuated from one to three (2.4 ± 0.8) times a week.	–
Farinha et al. (2018) [15], Brazil	Prior to randomization, individuals were required to adhere to their usual activity and dietary habits during a 4-week control phase.	Body weight (kg): ST (MD=1.2, ES=0.11), HIIT (MD=0.9, ES=0.08), ST+HIIT (MD=0.3, ES=0.02).
	The program has a total of 10 training sessions, which are to be held three times a week at the gym of the university. It is advisable to plan the sessions on days that are not consecutive. The initial two weeks of the program are specifically tailored to facilitate participants' progressive adjustment to the training procedure.	BMI (kg/m²): ST (MD=0.4, ES=0.13), HIIT (MD=0.3, ES=0.07), ST+HIIT (MD=0, ES<0.01).
	HIIT arm: involved using a cycling ergometer for 10 sets of 60-second intervals. The goal was to reach 90% of HRmax. After each interval, there was a 60-second active recovery period. Each session had a duration of 25 minutes, consisting of a 3-minute warm-up and a 2-minute cool-down at 50% of the HRmax.	Total body lean mass (kg): ST (MD=1.9, ES=0.40), HIIT (MD=0.9, ES=0.09), ST+HIIT (MD=1.4, ES=0.22).
	ST arm consisting of the following exercises: bench press, leg press, lat pulldown, leg extension, shoulder press, and leg curl. Participants were instructed to complete eight repetitions per set using proper technique and the maximum weight they were capable of handling. Additionally, three sets of abdominal crunches, each consisting of 15 repetitions, were performed. Each series was followed by a 1-minute rest interval, resulting in a total of 40 minutes of strength exercise.	Total body fat mass (kg): ST (MD=-1.3, ES=0.26), HIIT (MD=-0.2, ES=0.04), ST+HIIT (MD=-1.5, ES=0.20).
	The ST+HIIT arm: workout involved a series of consecutive ST and HIIT exercises, lasting approximately 65-70 minutes in total.	–
	All HIIT and ST+HIIT sessions were supervised by exercise physiologists and used Polar Electro Oy, Kempele, Finland heart rate monitors. Each workout ended with a regular stretch.	–
Jewiss et al. (2017) [16], Australia	In the studies, interventions included either aerobics, strength, or both types of exercise.	Body mass (kg) for adults: MD=-2.20 kg; 95% CI -3.79, -0.61; P=0.007. Body mass (kg) for children: MD=0.95 kg; 95% CI: 0.17, 1.73; P=0.02. Body mass (kg) post-2000 studies: MD=0.54; 95% CI: -2.1, 1.02; P=0.5.
	There were a total of 596 participants, with 360 in the exercise group and 236 in the control group. Out of the total studies, five involved adults and six were completed prior to 2000. The investigations include information gathered from a total of 9,251 hours of exercise training among the patients.	BMI (kg/m^2^) for adults: MD=-0.39 kg/m^2^; 95% CI: -0.75, -0.02; P=0.04. BMI (kg/m^2^) for children: MD=0.29 kg/m^2^; 95% CI: 0.03, 0.61; P=0.07. BMI (kg/m^2^) post-2000 studies: MD=-0.11 kg/m^2^; 95% CI: -0.43, 0.21; P=0.5.
	The studies varied in length, ranging from 12 to 26 weeks, with an average duration of 18.7 weeks and a median duration of 16 weeks. The number of exercise sessions per week ranged from 1 to 7, with a median of 3 sessions. The duration of each session ranged from 20 to 120 minutes, with a median duration of 47.5 minutes.	Waist circumference in children (cm): MD: -5.4 cm, 95% CI (-8.45, -2.35); p = 0.0005
Gusso et al. (2016) [17], New Zealand	The T1DM+ intervention arm and healthy control arm engaged in a 20-week training program, whereas the T1DM+ control arm did not.	Weight (kg): T1DM+I (MD=0.4, P<0.05), T1DM+C (MD=1.8, P<0.05), HC (MD=1.1, P>0.05).
	Four 60-minute workouts each week (including warm-up and cool-down) were done for 20 weeks. Individual or group sessions were supervised by an exercise physiologist.	BMI (kg/m2): T1DM+I (MD=0, P>0.05), T1DM+C (MD=0.4, P>0.05), HC (MD=0.1, P>0.05).
	Max HR: In weeks 1–4, aerobic exercise training was gradually altered to reach 85% of the participant's VO_2_ peak test heart rate. From weeks 5 to 20, participants were instructed to exercise at 85% of their maximum heart rate for at least 40 minutes every aerobic session.	Percent fat-free mass: T1DM+I (MD=1, P<0.05), T1DM+C (MD=-1.6, P>0.05), HC (MD=0.8, P<0.05).
	Exercise pattern: From weeks 1–12, participants were instructed to do at least three cardio workouts a week (treadmill, cycling, rowing machine, and interval training) followed by weekly resistance training (weightlifting and core exercises).	Percent total body fat: T1DM+I (MD=-1.1, P<0.05), T1DM+C (MD=1.3, P<0.05), HC (MD=-1.1, P<0.05).
	Participants did four aerobic and resistance workouts a week from weeks 12–20 called circuit training. This exercise routine alteration was intended to maintain participant involvement and maximize exercise protocol compliance. In between exercises, participants had 30 seconds to catch their breath.	–
	Each training group member received a downloaded heart rate monitor (Polar S625X; Polar Electro, Kempele, Finland) to track their workouts.	–

Recommendation From the American Diabetes Association Regarding PA

The American Diabetes Association advises that persons with T1DM engage in at least 150 minutes of moderate-to-vigorous aerobic activity per week. This should be spaced out over a minimum of three days, with no more than two consecutive days without PA. If the patient is in good physical condition, engaging in vigorous activity for a short period (75 minutes a week) may be enough. Children and adolescents should participate in PA for at least 60 minutes per day, at least three days per week, at a moderate-to-vigorous intensity. This should include exercises that strengthen muscles and bones. On the other hand, adults should engage in resistance exercise two to three times per week on non-consecutive days. Ultimately, it is recommended that older individuals engage in flexibility and balance training biweekly, such as practicing yoga and Tai Chi [7]. These are compatible with the type 2 diabetes guidelines.

Outcome of the Intervention

After the intervention, five studies observed a significant difference in body mass per kilogram (kg) [6,12,14,16,17]. Adults experienced a decrease in body weight; however, adolescents and children had an increase. There are two possible explanations for this difference: muscle gain or growth via increasing fat and fat-free mass [18,20]. Regarding BMI, most research [3,6,13,15,17] found no significant changes except for a systematic review conducted by Ostman et al. [16], which determined that adults had a lower BMI than children. In addition, Quirk et al. [12] found that the combined exercise group experienced a substantial reduction in BMI compared to the control group. It is noteworthy to note that losing weight significantly - rather than just concentrating on the number of kilograms lost - is necessary when using BMI as a measure.

Regarding muscle mass, there was a significant increase in total body lean mass by 2 kg in the strength exercise group. This was compared to 0.9 kg in the high-intensity interval training (HIIT) group and 1.4 kg in the combined arm [15]. This is consistent with Gusso et al.'s [17] findings, which concluded that the fat-free mass of T1DM patients with intervention increased by 1% and that of healthy controls by 0.8%, while the fat-free mass of the controls for the patients with T1DM decreased by 1.6%. Wrobel et al. [3] concluded that resistance training did not significantly increase muscle mass among T1DM patients. However, it increased by 0.7 kg compared to no increase in the aerobic training group. In addition, Mosher et al. [23] determined that adolescent males with T1DM gain lean body mass following aerobic circuit exercise training. Therefore, we can conclude that T1DM patients must include resistance exercise in their activity schedule. This is because increasing muscle mass can improve BMI in underweight patients and reduce insulin doses, blood sugar, glycemic value, HbA1c, and cholesterol levels [23,24]. Furthermore, research revealed that extending the duration of moderate, vigorous, or moderate-to-vigorous PA led to an increase in fat-free mass [20,21]. On the other hand, training in hypoxic environments leads to greater muscle hypertrophy, but this effect was not significant according to Wrobel et al., [13] who explained it as a result of muscle structure distortion caused by diabetes.

Most studies reported fat mass as one of their outcomes. Furthermore, total body fat mass decreased by 1.3 kg in the strength exercise group and 1.5 kg in the combined group, which had strength and HIIT activity. However, it decreased only by 0.2 in the HIIT group [15]. Furthermore, the total body fat changed significantly, reducing by 1% in the T1DM and healthy control groups compared to an increase of 1.3% in the T1DM group [17]. Nevertheless, Wrobel et al. [3] concluded that the fat mass in the resistance group dropped by 0.9 kg in three months, while it increased by 0.7 kg in the aerobic group. However, this difference was not significant. Several factors, such as the duration, intensity, and health status of the patients, may account for this difference. However, T1DM patients have demonstrated the crucial role of strength training in reducing body fat. Sideraviciūte et al. [19] found that adolescents with T1DM experienced a decrease in fat mass following participation in a swimming program as an intervention. Hence, swimming is a dual-purpose activity, encompassing both aerobic and resistance components simultaneously.

Personalized Exercise Prescription Approaches for Underweight T1DM Patients

The recommendations from diabetes guidelines were focused primarily on glycemic control and prevention of cardiovascular complications. However, exercise has a major role in T1DM management, specifically if it is personalized to each individual using body composition analysis, continuous glucose monitoring, and hormonal evaluation [25,26].

The exercise prescription for underweight T1DM patients should limit excessive energy expenditure and focus on anabolic stimuli. Furthermore, Yardley et al. [27] found that to maximize muscle protein synthesis and permit adequate recovery for T1DM patients, exercise sessions should be planned as three consecutive resistance sessions per week.

On the other hand, meal consumption and insulin administration timing should be carefully administered. A T1DM patient should take a carbohydrate snack before exercise (1-1.5 g/kg consumed 1-2 hours before activity) and reduce rapid-acting insulin by 25-50% to support the anabolic process and minimize the hypoglycemia risk during resistance exercise [26,28].

Based on a community-based approach, Faulkner et al. [29] implemented a novel intervention that included adolescents with T1DM engaging with a certified personal trainer in twice-weekly supervised sessions and home-based activities. He concluded that moderate-to-vigorous PA is linked to enhancing glycemic control by reducing HbA1c levels even if they did not reach the recommended time (60 minutes of moderate-to-vigorous PA).

Application of PA Recommendation in Lifestyle Medicine Clinics

Parents exert the greatest influence on children's behavior about PA. Parents have a crucial role in facilitating their children's PA experiences [30]. Consequently, parents can shape their children's PA by demonstrating positive attitudes towards a healthy lifestyle [31,32]. Offering support and acknowledging their efforts have a significant impact on a child's self-efficacy, which is widely recognized as a crucial factor in motivating the youngster [33,34]. Parental support, achieved via thoughtful planning and active participation in PAs, plays a crucial role in helping children overcome obstacles in engaging in PA [35]. Despite encountering barriers such as activity planning, blood glucose control to avoid hypoglycemia, and the need for a strong support system, parents have noticed the beneficial impacts of PA on their children. These benefits include body composition enhancements, blood glucose control, and understanding how the body reacts to food, exercise, and insulin [36].

Addressing stigma among adolescents with T1DM necessitates the cultivation of personal and community knowledge regarding the condition [36]. Engaging in PA will be facilitated by T1DM patients' personal insulin pharmacokinetic knowledge [37]. Additionally, it assists them in managing their stress [38]. Teachers and coaches must acquire knowledge about the disease from T1DM patients, as they are experts, as this will reduce stigmatization [39]. Adolescents with T1DM may be discouraged from participating in team-based PA with their peers due to feelings of stigmatization [40]. Therefore, enhancing the level of personal interaction between individuals with T1DM and others has the potential to increase empathy and mitigate negative stereotypes [41]. The potential to reduce stigma is present when adolescents are involved in the formulation and implementation of training programs for the community and their peers. Furthermore, adolescents with chronic diseases are influenced by their social connections in terms of their quality of life and stigma [42].

Support and guidance from healthcare professionals are critical in managing the hypoglycemia risk and promoting self-efficacy in people with T1DM [43]. Self-efficacy refers to a person's belief in their ability to successfully carry out specific behaviors [44]. Healthcare workers should work as a team to care for T1DM patients, taking safety precautions during training in various settings such as schools, universities, and homes [45]. Additionally, parents and peers can have a positive influence on supporting individuals with T1DM [46,47]. It is important for healthcare providers to have knowledge and competence in managing T1DM, especially when supervising children's PAs. Working together with families can ensure a thorough understanding of managing PA in children with T1DM rather than solely focusing on activity promotion [45]. These findings help educate professionals about the difficulties patients with T1DM encounter.

A staff member with specific training in providing PA advice and guidance could greatly benefit diabetes teams [45]. This individual should possess the necessary qualifications and training in diabetes management, allowing them to take a holistic approach when working with diabetic patients.

Using a PA log sheet or applications to track the PA level might help patients for patients and health care providers adjust the treatment dose. In addition, they might modify the exercise plan according to their needs. Furthermore, we need to develop a DM support community to engage patients and their relatives more in educational sessions, as well as increase PA programs in the community.

## Conclusions

Resistance exercise increased muscle mass and reduced fat mass in T1DM patients, improving BMI and other health markers. Increasing the duration and intensity of PA also led to an increase in fat-free mass. Parental support and positive attitudes towards a healthy lifestyle can motivate children to engage in PA. Addressing stigma among adolescents with T1DM is important, and personal and community knowledge about the condition can help facilitate PA participation. Healthcare professionals should provide support and guidance to manage hypoglycemia risk and promote self-efficacy in T1DM patients. Collaboration between healthcare providers, families, and peers is essential in managing PA in T1DM patients. Having a staff member with specific training in providing PA advice can benefit diabetes teams.
